# Dissecting the genetic architecture of yield-related traits by QTL mapping in maize

**DOI:** 10.3389/fpls.2025.1624954

**Published:** 2025-08-15

**Authors:** Hao Zhang, Ting Li, Zhenyu Zhang, Jie Wang, Haoxiang Yang, Jiachen Liu, Wanchao Zhu, Jiquan Xue, Shutu Xu

**Affiliations:** ^1^ Hainan Institute of Northwest A&F University, Sanya, Hainan, China; ^2^ The Key Laboratory of Maize Biology and Genetic Breeding in Arid Area of Northwest Region, College of Agronomy, Northwest A&F University, Yangling, Shaanxi, China

**Keywords:** maize, yield, QTL mapping, recombinant inbred line population, candidate genes

## Abstract

**Introduction:**

Maize is a cornerstone of global agriculture, essential for ensuring food security, driving economic development, and meeting growing food demands. Yet, how to achieve optimal yield remains a multifaceted challenge influenced by biotic, environmental, and genetic factors whose comprehensive understanding is still evolving.

**Methods:**

QTL mapping of eight essential yield traits was conducted across four environments — Sanya (SY) in 2021, and Yangling (YaL), Yulin (YuL), and Weinan (WN) in 2022 — using two types of populations: a KA105/KB024 recombinant inbred line (RIL) population and two immortalized backcross populations (IB1 and IB2) derived from the RILs by crossing with their respective parents. Key candidate genes were identified through the integration of RNA-seq data, gene-based association analysis and classic yield-related genes network dataset.

**Results:**

Greater phenotypic variation was observed in RIL population than that in the IB1 and IB2 populations, while similar phenotype variations between IB1 and IB2 populations. A total of 121 QTLs were identified, including 10 QTLs that regulate multiple traits and 41 QTLs shared among these populations. Notably, 59.5% of the 42 QTLs identified in the IBL population (combined mapping using populations IB1, IB2, and RIL) exhibited an overdominance effect through the simultaneous calculation of additive and dominant effects. Through integrated transcriptome data and interaction networks, 20 genes located in these QTLs were investigated as candidate genes. Among them, *Zm00001d005740* (*ZmbHLH138*) was significantly associated with ear diameter in the association mapping panel AM508.

**Conclusion:**

These findings illuminate the genetic mechanisms underpinning maize yield formation, providing a robust foundation for advancing high-yielding variety development through targeted field breeding strategies.

## Introduction

Maize (*Zea mays* L.) is one of the most important crops worldwide for its extensive cultivation, high grain yield, and widespread use of hybrid varieties. As a crop with combined value for food, feed, and industrial use, maize plays a vital role in ensuring food security, supporting livestock development, and supplying raw materials for industry, thereby contributing positively to improving household income and living standards. The latest forecast indicates that global maize production is expected to increase by 3.8% in 2025, with a projected 6.0% rise in the United States (FAO, 2025). In China, maize is cultivated over an area of 44.74 million hectares, with a total output of 294.92 million tonnes, accounting for 41.7% of the country’s total grain production (National Bureau of Statistics of China, 2025). The yield of maize is mainly determined by the ear number per unit area, the number of kernels per ear and hundred kernel weight (HKW). Among them, the HKW is related to the process of kernel filling and kernel traits, while the number of kernels per ear is related to the ear traits. Kernel traits mainly include kernel thickness (KT), kernel length (KL), kernel width (KW), and HKW. The ear traits mainly include ear length (EL), diameter (ED), ear row number (ERN), kernel number per row (KNR), etc (Dong et al., 2023).All of these traits are associated with yield, with broad-sense heritability (*H²*) ranging from 0.61 to 0.83 (Zeng et al., 2022).

Numerous studies have focused on investigating the complexities of yield and its associated traits (Ma and Cao, 2021; Luo et al., 2022; Qian et al., 2023). Phenotypic and genetic correlation analyses show that component traits EL, ED, ERN, KNR, KL, KW, grain yield per plant (GYP), and HKW are associated with yield (Shi et al., 2017). Notably, KL, KW, HKW, EL, KNR, and ear weight (EW) show positive correlations with individual plant yield (Li et al., 2013; Yang et al., 2016; Xiao et al., 2022). Meanwhile, the relationship between yield-associated traits in maize must also be considered. One study identified a significant positive correlation between ED and ERN, and a slight negative correlation between KNR and HKW (Zhang et al., 2020). Another study demonstrated that HKW, KL, KW, and KT had a significant positive correlation, while KT exhibited a strongly negative correlation with KL (Lan et al., 2018). More recent work has also shown that yield is positively correlated with plant height and ear height under various environmental conditions. Interestingly, while the interval between flowering and silk emergence showed no correlation with yield under irrigation conditions, it was significantly negatively correlated under drought stress conditions (Xue et al., 2013). These studies collectively indicate that yield is influenced by multiple interconnected component traits, highlighting the importance of exploring relationships among different traits, identifying functional loci and genes, and elucidating regulatory mechanisms to improve yield.

Over the years, researchers have developed a variety of populations to dissecting the genetic architecture of yield-related traits, such as recombinant inbred line (RIL), F_2_ population, doubled haploid (DH) population, backcross (BC) population, and immortalized backcross (IB) populations (Wu et al., 2016; Aakanksha et al., 2021; Mei et al., 2021; Chen et al., 2024; Han et al., 2025). RIL is a type of permanent mapping population developed by repeated selfing from an F_2_ generation, typically for more than six generations (Takuno et al., 2012). As a result, each line becomes nearly homozygous, yet carries a unique combination of recombined parental alleles. Due to their genetic stability, RILs can be repeatedly phenotyped across different environments and years, making them highly suitable for dissecting the genetic basis of complex traits such as yield (Hu et al., 2020). Although RIL populations offer many advantages for dissecting the genetic architecture of yield-related traits, they are limited to detecting additive effects. However, dominance effects also play a critical role in understanding the inheritance of these traits. IB populations are a type of specialized genetic population developed by backcrossing a segregating population, such as RIL, to both parental lines, resulting in hybrid progeny suitable for dominant effect analysis (Huo et al., 2023). Therefore, the combination of RIL and IB populations is particularly useful for detecting both additive and dominant genetic effects, making them powerful tools for dissecting the genetic architecture of yield-related traits.

Yield-related traits in maize are usually complex quantitative traits. To dissect their genetic architecture, linkage mapping using various bi-parent mapping populations has identified a handful of quantitative trait loci (QTL) for both ear traits and kernel traits (Wang et al., 2019; Ning et al., 2021; Li et al., 2023). For instance, a F_2:3_ populations from parents Zong-3 and 87–1 was utilized to identify 29 QTLs related to yield, including kernel number per row (KNR), hundred kernel weight (HKW), and ear row number (ERN) (Yan et al., 2006). Similarly, Huo et al. detected 39 QTLs associated with maize ear traits (Huo et al., 2016), and Liu et al. identified 33 QTLs for the ERN (Liu et al., 2015). Zhou et al. revealed 14 QTLs for EL using an F_2:3_ population derived from Zheng58 × Chang7–2 and a four-cross population (Zhou et al., 2018). Three QTLs were reported for EL and ERN in an F_2_ population derived from Chang7-2 × 787 (Chen et al., 2014b), and another 6 ear trait loci were identified in a mapping study with KA105 × KB020 F_5:6_ families (Yang et al., 2021). These findings indicate that yield-related traits are mainly controlled by rare major QTLs and many minor QTLs.

In recent years, significant progress has been made in the molecular cloning of genes that influence maize yield, particularly those influencing ear and kernel development (Li et al., 2021; An et al., 2022; Chen et al., 2023). For instance, *Zm00001d022088* (*ZMM28*), a MADS-box transcription factor, has been found to positively influence growth, photosynthetic efficiency, and ultimately yield when overexpressed (Wu et al., 2019). Similarly, *Zm00001d027877* (*ZmBG1H1*), a member of the BG1 family, is associated with increased ERN and grain yield. However, its overexpression results in a reduction in grain size (Simmons et al., 2020). Another notable gene is *Zm00001d002641* (*KRN2*), which encodes a WD40 family protein and plays a role in determining maize ear row number (Chen et al., 2022). Knocking out *KRN2* increases ERN without compromising other agronomic traits, resulting in a notable 10% increase in maize yield. Additionally, *Zm00001d017570 EAD1*, an aluminum-activated malic acid transporter, has been linked to enhanced EL and KNR (Pei et al., 2022). Furthermore, genes like *ZmGW2*, which encodes a RING-type E3 ubiquitin ligase, have been found to influence KW and HKW (Li et al., 2010). These advancements in understanding and manipulating key genes such as *ZmM28*, *ZmBG1H1*, *KRN2*, *EAD1*, and *ZmGW2*, along with the potential discovery of new genes, hold significant promise for further enhancing maize yield through targeted genetic interventions.

Yield-related traits belong to complex quantitative traits, controlled by multiple minor genes and significantly influenced by environmental factors. Although traditional breeding has led to the development of many high-yielding varieties, its detailed genetic mechanism still not clear, which limited improved it by molecular biochemical technology. Understanding the genetic basis of yield-related traits is therefore essential to accelerate genetic improvement. To address this challenge, we employed a RIL population and two immortalized backcross populations (heterozygosis population) to dissect the genetic architecture of eight key yield-related traits. By leveraging multiple populations across diverse environments, we enhanced the robustness and generalizability of our findings while capturing a broader spectrum of genetic variation. The integration of both additive and dominant genetic models enabled a more comprehensive understanding of trait inheritance and facilitated the identification of QTLs and candidate genes. Nevertheless, potential environmental interactions may not have been fully accounted for, and further functional validation of candidate genes is still required.

## Materials and methods

### Plant materials

A RIL population with 183 lines was developed from two inbred lines KA105 and KB024 to detect QTLs for yield-related traits. In May 2021, KA105, KB024, and the 183 RILs were cultivated at the Yangling maize base of Northwest A&F University. Subsequently, each of the 183 RILs was backcrossed with their parents KA105 and KB024, respectively, to develop two IB populations, each consisting of 183 BC_1_ progeny (Yang et al., 2024). The IB population derived from backcrossing with KA105 was designated as the IB1 population, while the counterpart obtained from backcrossing with KB024 was labeled as the IB2 population, the entirety population including IB1, IB2, and RIL is named IBL (Zhang et al., 2022).

### Plant growth and trait measurements

The experiment was conducted across four environments: Sanya (SY) in November 2021, Yangling (YaL) and Yulin (YuL) in May 2022, and Weinan (WN) in June 2022. It utilized a randomized block design with a row spacing of 0.6 meters, a single row with 4 meters for row length and 0.22 meters for plants interval. Field management practices strictly adhered to local protocols. At maturity, all ears within the experimental plots were harvested, and five representative ears were selected for comprehensive trait analysis, including ED, EL, ERN, and KNR. These chosen ears were subsequently sun-dried naturally and threshed, followed by weighing to determine GYP. Additionally, 100 seeds were randomly sampled and weighed, with this procedure replicated thrice for precision. Lastly, KL and KW were assessed using a multifunctional digital corn seed testing machine (iMaize, Phenotrait).

### Phenotypic data analysis

R package “psych” (https://cran.r-project.org/web/packages/psych/) was used for basic descriptive statistics (Revelle and Revelle, 2015). R package “corrplot” (https://cran.r-project.org/web/packages/corrplot/) was used to calculate the correlation coefficient between traits (Wei et al., 2017). R package “lme4” (https://cran.r-project.org/web/packages/lme4/) calculated broad sense heritability (*H^2^*) using this formula (Bates et al., 2015):


(1) $$H^{2}=\frac{{\text{σ}}_{\text{g}}^{2}}{\left({\text{σ}}_{\text{g}}^{2}+\frac{{\text{σ}}_{\text{e}}^{2}}{\text{n}}\right)}$$

Where ${\text{σ}}_{\text{g}}^{2}$ is the genotype variance, ${\text{σ}}_{\text{e}}^{2}$ is the environment variance, and n is the number of environments.

### Linkage map construction

In total, 183 leaf samples from RILs and parental lines were collected from the field for genomic DNA extraction and subsequent genotype detection (Yang et al., 2024). DNA extraction followed an improved CTAB method known for its efficacy in yielding high-quality samples. The extracted DNA was genotyped using the Maize 6H-60k SNP chip developed at Beidahuang Kenfeng Seed Co., Ltd., using the maize inbred line B73 version 3 (AGPv3) genome as a reference from the Maize Research Center of Beijing Academy of Agriculture and Forestry Sciences. The genetic map construction involved the following steps: 1) Screening SNP markers for polymorphism in both KA105 and KB024. 2) Using the QTL IciMapping (v4.2) software’s “BIN” function to identify effective markers. 3) Marker selection criteria included a missing rate ≤10% and a distortion value P ≥ 0.0001. 4) Generating a genetic map using the “MAP” function with threshold value = 0.3 and window size = 5.

### QTL mapping

QTL mapping for each trait was conducted in the inclusive composite interval mapping (ICIM) mode on the “QHP” function of GAHP (v1.0) (Zhang et al., 2022). The LOD value was determined using 1000 permutations, which were 3.58, 3.59, 3.62, and 3.95 for RIL, IB1, IB2, and IBL, respectively. The type I error rate set to 0.05 and a PIN threshold of 0.001. This software enables simultaneous QTL mapping across the RIL population, IB1 and IB2 (two permanent backcross populations), and IBL (integrated RIL and backcross populations). QTL detection in the RIL population calculates additive effects (a). In IB1 and IB2 populations, it calculates a-d and a+d effect, respectively, where d represents dominant effects. QTL detection in the IBL population distinguishes both additive (a) and dominant (d) effects simultaneously (Huo et al., 2023). QTLs in the IBL population were categorized based on their degree of dominance d/a as follows: additive effects (A) range from 0.00 to 0.20, partial dominance (PD) from 0.21 to 0.80, dominance (D) from 0.81 to 1.20, and overdominance (OD) above 1.20 (Edwards et al., 1987). Consistency in QTL intervals and effects identifies a single QTL. A QTL explaining over 10% of phenotypic variance (PVE) is classified as a major QTL.

### Yield-associated candidate gene analysis

QTLs with PVE > 10% that co-locate across different environments, populations, or traits are classified as major QTLs. All genes within the QTL interval are identified according to the B73 reference genome. Integrated network datasets and two public transcriptome datasets of B73 were downloaded from the National Center for Biotechnology Information, including kernel (embryo, endosperm, and whole seed) and cob (immature and pre-pollination stages) at various time points (accession numbers: PRJNA171684, PRJEB10574, PRJNA226757, PRJNA244661, PRJNA323555, PRJNA369690, and SRP037559) (Chen et al., 2014a; Hoopes et al., 2019; Feng et al., 2023; Han et al., 2023). Genes with FPKM > 5 were considered highly expressed genes of interest. Genes expressed in kernel and cob were compared with QTLs associated with kernel and cob to determine candidate genes. Gene-based association analysis was performed to further identify key gene in a classical association mapping panel consisting of 508 diverse maize inbred lines (Yang et al., 2014). The genotypes and phenotypes were downloaded from the Maizego website (www.maizego.org), where the genotypes included polymorphic loci spanning gene region and 2 kb upstream and downstream, the phenotype was the Best Linear Unbiased Estimate (BLUE) value. Association mapping and linkage disequilibrium were performed using TASSEL (v5.0) and LDBlockShow (Bradbury et al., 2007; Dong et al., 2021). Moreover, the co-expression network between the key gene and the known yield trait-associated genes was constructed, which was visualized with Gephi (V0.9.2) (Bastian et al., 2009). Gene Ontology (GO) analysis was performed using the agriGO web server (http://bioinfo.cau.edu.cn/agriGO/index.php) with a p-value ≤ 0.01 and false discovery rate (FDR)< 0.05 (Tian et al., 2017).

## Results

### Phenotypic variation in yield-related traits

**Figure 1:**
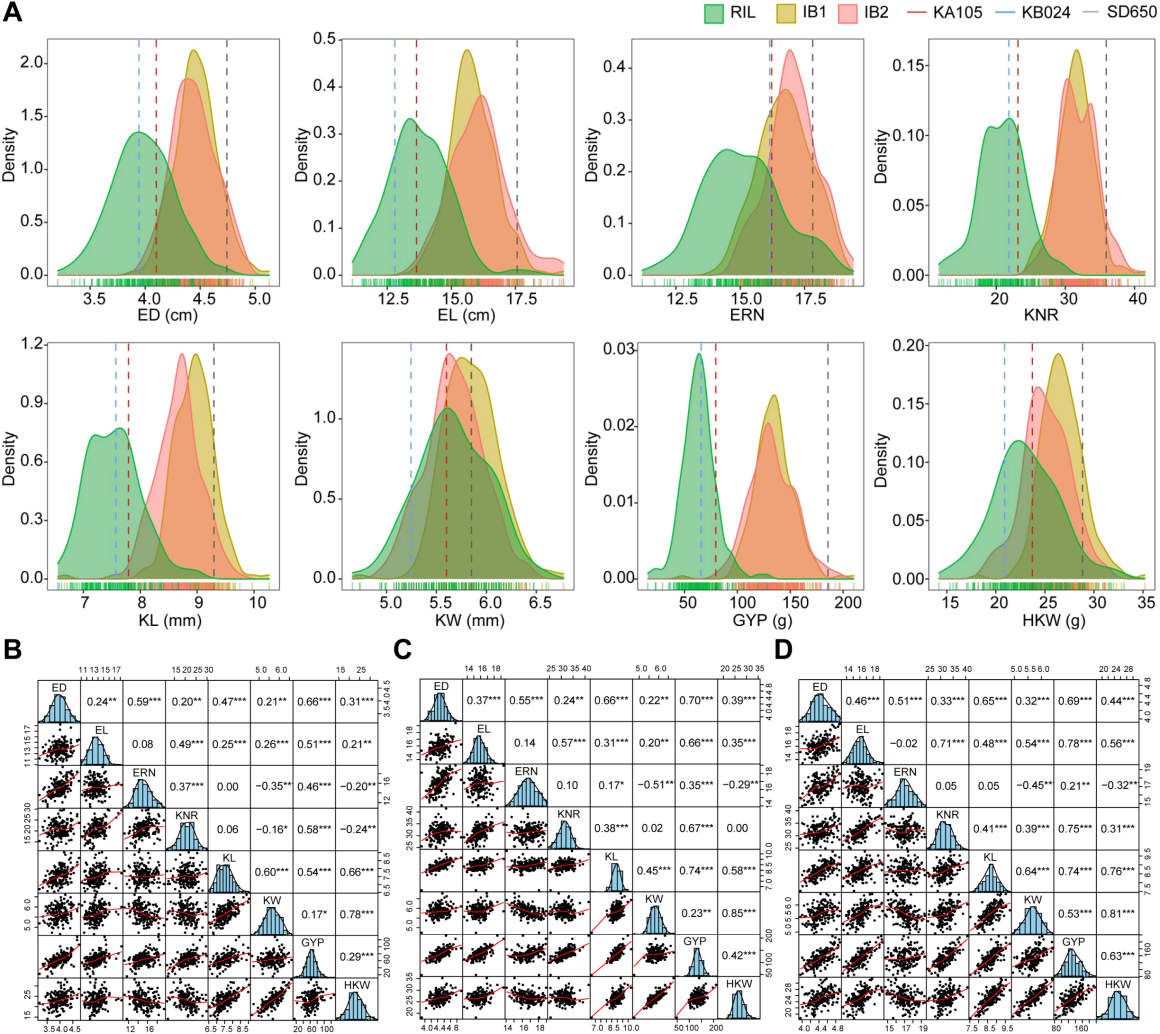
Analysis of phenotype and correlations in different populations. **(A)** Distribution of best linear unbiased estimation (BLUE) for the eight yield traits in the RIL, IB1, and IB2 populations. **(B)** Correlations in the RIL population. **(C)** Correlations in the IB1 population. **(D)** Correlations in the IB2 population. ***, **, and * refer to significant at 0.001,0.01,and 0.05 level, respectively.

Here, eight yield-related traits (ED, EL, ERN, KNR, KL, KW, GYP, HKW) were investigated for RIL, IB1, IB2, as well as the two parents (KA105, KB024) and the related F_1_ (**Supplementary Figure S1**). Except for ERN, the BLUE values of all traits in KA105 were significantly higher than those in KB024 (**Figure 1A**; **Supplementary Table S1**). Both the RIL and the two backcross (IB1 and IB2) populations displayed abundant phenotypic variation across all four environments, where the coefficient of variation (CV) varied ranging from 4% to 55%. Generally, the CV for each trait in the RIL population was higher compared to the IB1 and IB2 populations, with no significant difference observed between IB1 and IB2. This might be due to the existence of heterozygous genotypes in the backcross population, which reduced the phenotypic variation.

Additionally, *H^2^* of all traits were calculated (Equation 1), which were ranged from 0.55 to 0.73 in the RIL population, 0.51 to 0.89 in IB1, and 0.71 to 0.88 in IB2. Most traits in IB1 and IB2 populations showed slightly higher *H^2^* values than those in the RIL population. Overall, the high heritability illustrated that all studied traits were influenced by genotype. Correlation analysis revealed highly significant correlations among most traits within each of the three populations, suggesting potential synergistic regulation (**Figures 1B–D**).

### Genetic linkage map and QTL mapping

**Figure 2:**
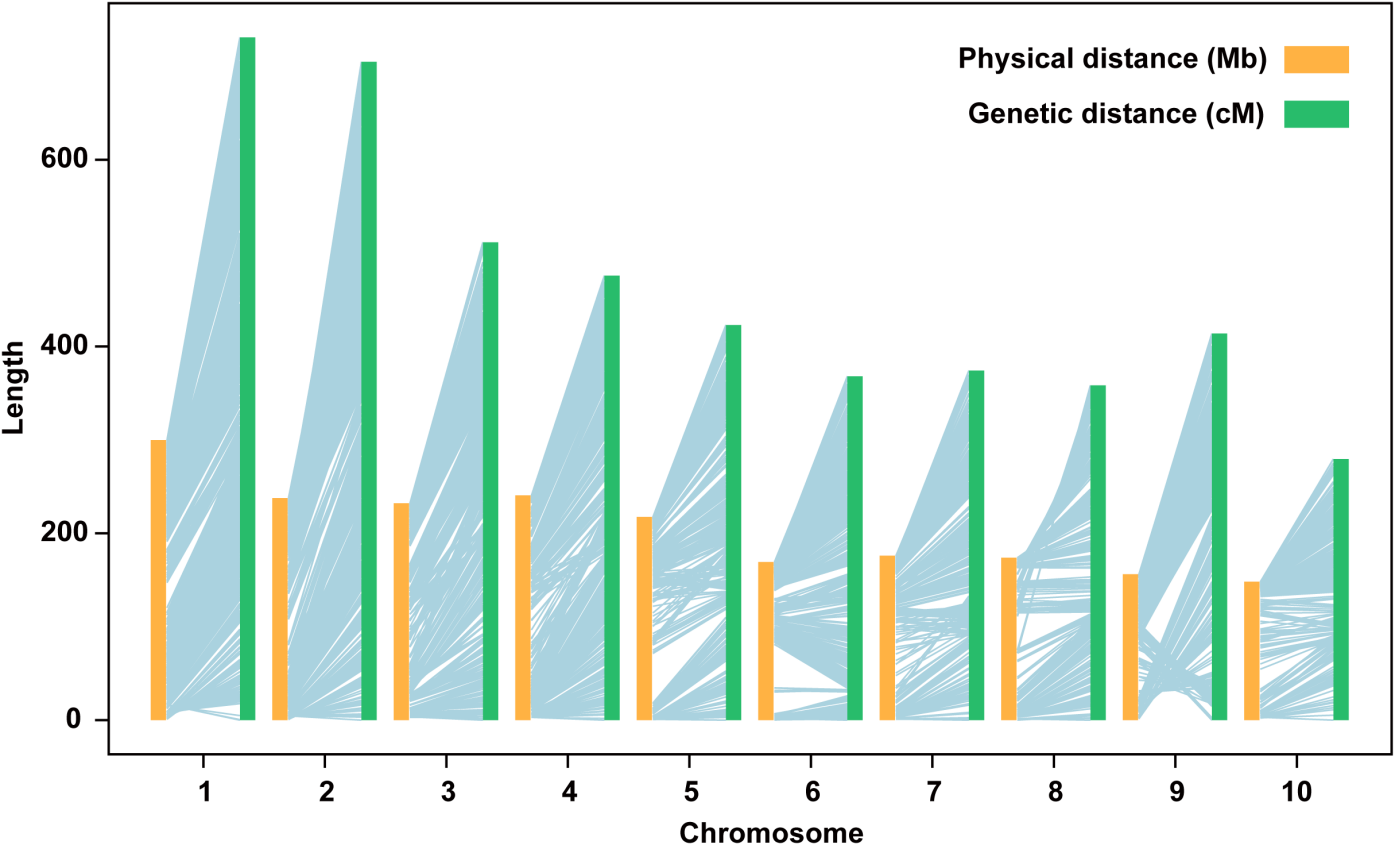
Collinearity between the genetic map of the RIL population and the physical map of the B73 (AGPv3) genome.

**Figure 3:**
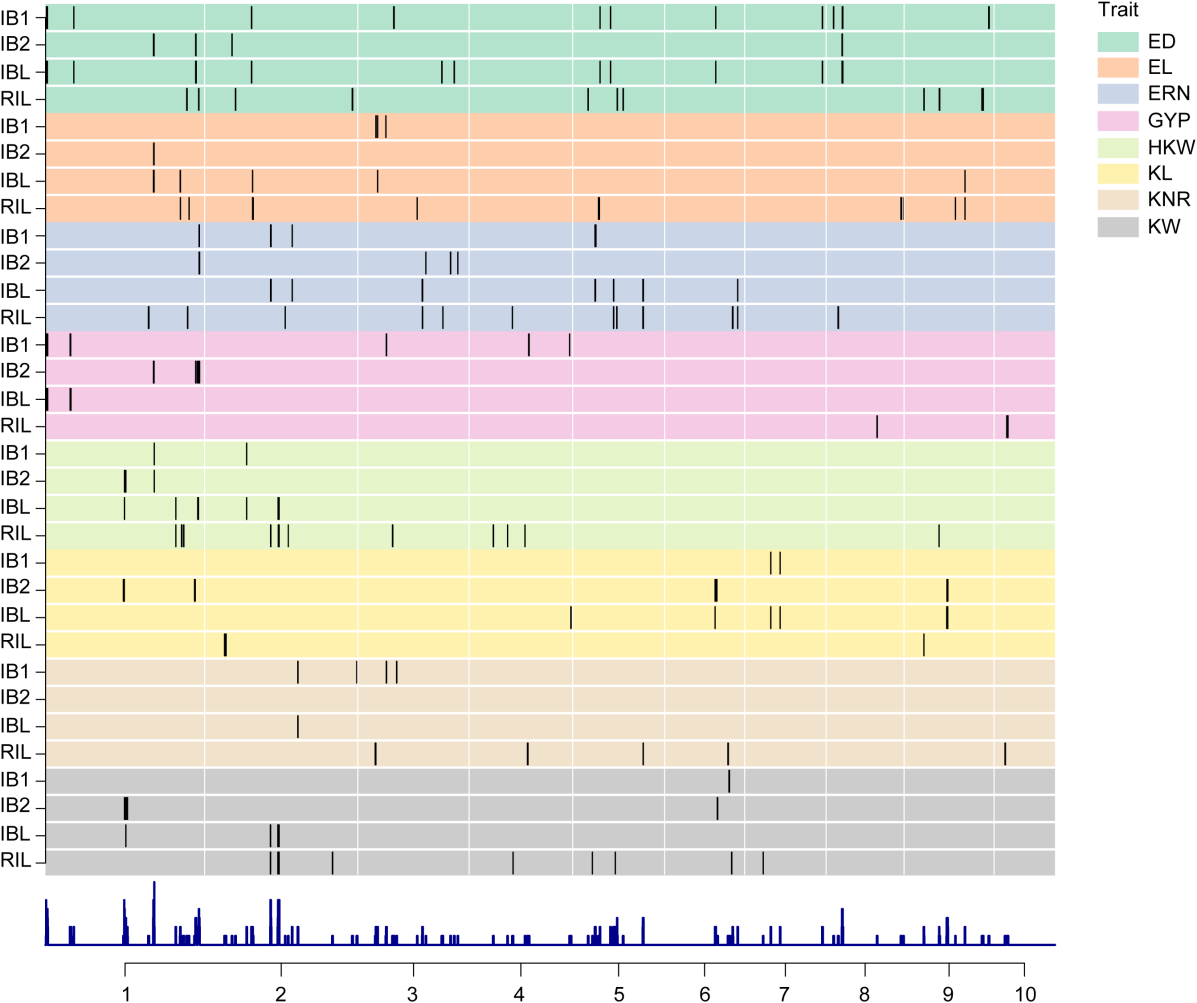
Distribution of QTLs associated with the eight yield traits across different populations. ED, ear diameter; EL, Ear length; ERN, Ear row number; GYP, Grain yield per plant; HKW, hundred kernel weight; KL, Kernel length; KNR, Kernel number per row; KW, Kernel width. Each black vertical line represents a QTL position on the maize genome, grouped by population type and trait. The histogram at the bottom illustrates the overall QTL density across the maize genome.

A total of 4,555 high-quality SNPs were used for construction the genetic linkage map with an average of 1.04 cM interval, which have been described in previous study (Yang et al., 2024). Each chromosome containing at least 248 and at most 775 markers (**Supplementary Table S2**). There was high degree of collinearity between the genetic linkage map and the B73 (AGPv3) reference physical map, except for several inversions, especially on the short arm of chromosome 9 (**Figure 2**). Based on the genetic linkage map and phenotype, QTL mapping of eight yield-related traits was performed in RIL, IB1, IB2, and IBL populations. In total, 121 unique yield-related QTLs were identified (**Figure 3**).

**Figure 4:**
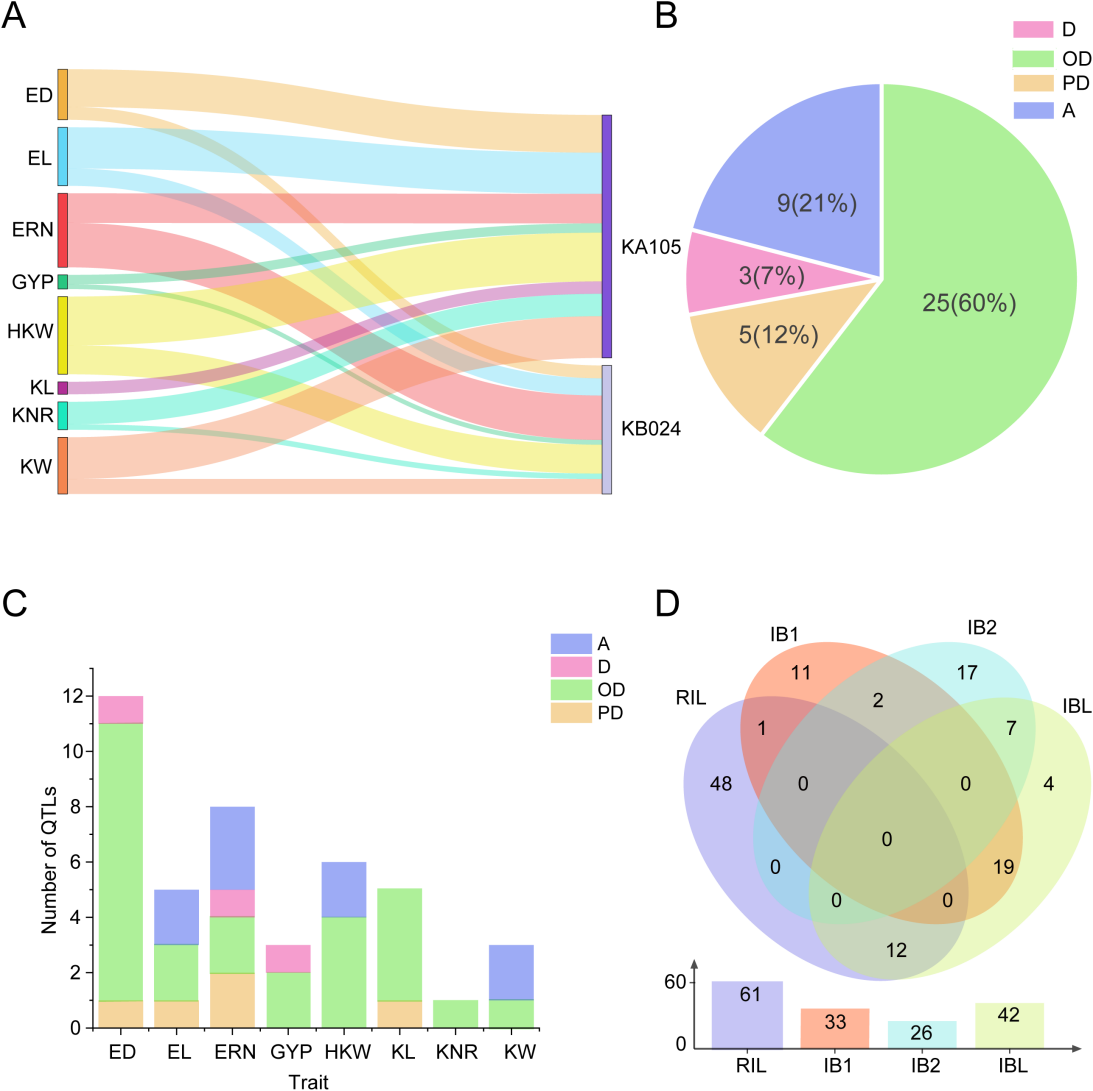
Analysis of QTLs from the RIL, IB1, IB2 and IBL populations. **(A)** Distribution of additive QTLs in the RIL population. **(B)** Proportions of QTLs exhibiting different effects in the IBL population. OD: over-dominance effect, D: dominance effect, PD: partial dominance effect, A: additive effect. **(C)** Numbers of QTLs exhibiting the PD, OD, D, and A effects for different yield traits in the IBL population. **(D)** Co-localization of QTLs detected across different populations.

In the RIL population, 61 QTLs were identified with a single QTL explaining 1.73% to 25.30% of phenotypic variation, and 11 QTLs of them with PVE greater than 10% (**Supplementary Table S3**). Among these 61 QTLs, 37 (60.66%) of the favorite alleles, associated with an improved phenotype were originated from KA105 (**Figure 4A**). Additionally, four QTLs (*qHKW2-3*, *qKW5-3*, *qERN5-3*, and *qERN6-1*) were consistently detected across different environments, indicating their environmental stability.

In the IB1 population, 33 QTLs were detected with 2.57% to 15.65% PVE for each QTL, and eight of them can explain more than 10% PVE (**Supplementary Table S3**). Among these, *qED1–1* was considered as environmentally-stable QTLs, which can be identified in two or more data sets. In the IB2 population, 26 QTLs were detected with 4.30% to 13.23% PVE. Notably, 17 QTLs were concentrated on chromosome 1 (**Supplementary Table S3**), of which, six were major QTLs and two QTLs (*qEL1–1* and *qKL9-2*) were environmentally stable.

In IBL, a total of 42 QTLs were detected with 0.43% to 6.11% PVE. *qHKW1–1* can be detected in two environments. In addition, several sets of QTLs colocalized to the same interval: *qGYP1–1* and *qED1-1*; *qHKW2–3* and *qKW2-2*; and *qKW2–1* and *qERN2-1* (**Supplementary Table S3**). Furthermore, genetic effect analysis revealed that 25 of those QTLs identified in IBL exhibited OD effect, 3 QTLs showed D effect, 5 QTLs showed PD effect, and 9 QTLs displayed A effect (**Figure 4B**). Twenty-four OD effect QTLs were detected for ED, EL, ERN, GYP, HKW and KL (**Figure 4C**), indicating that the OD effect mainly affects these yield-related traits.

**Figure 5:**
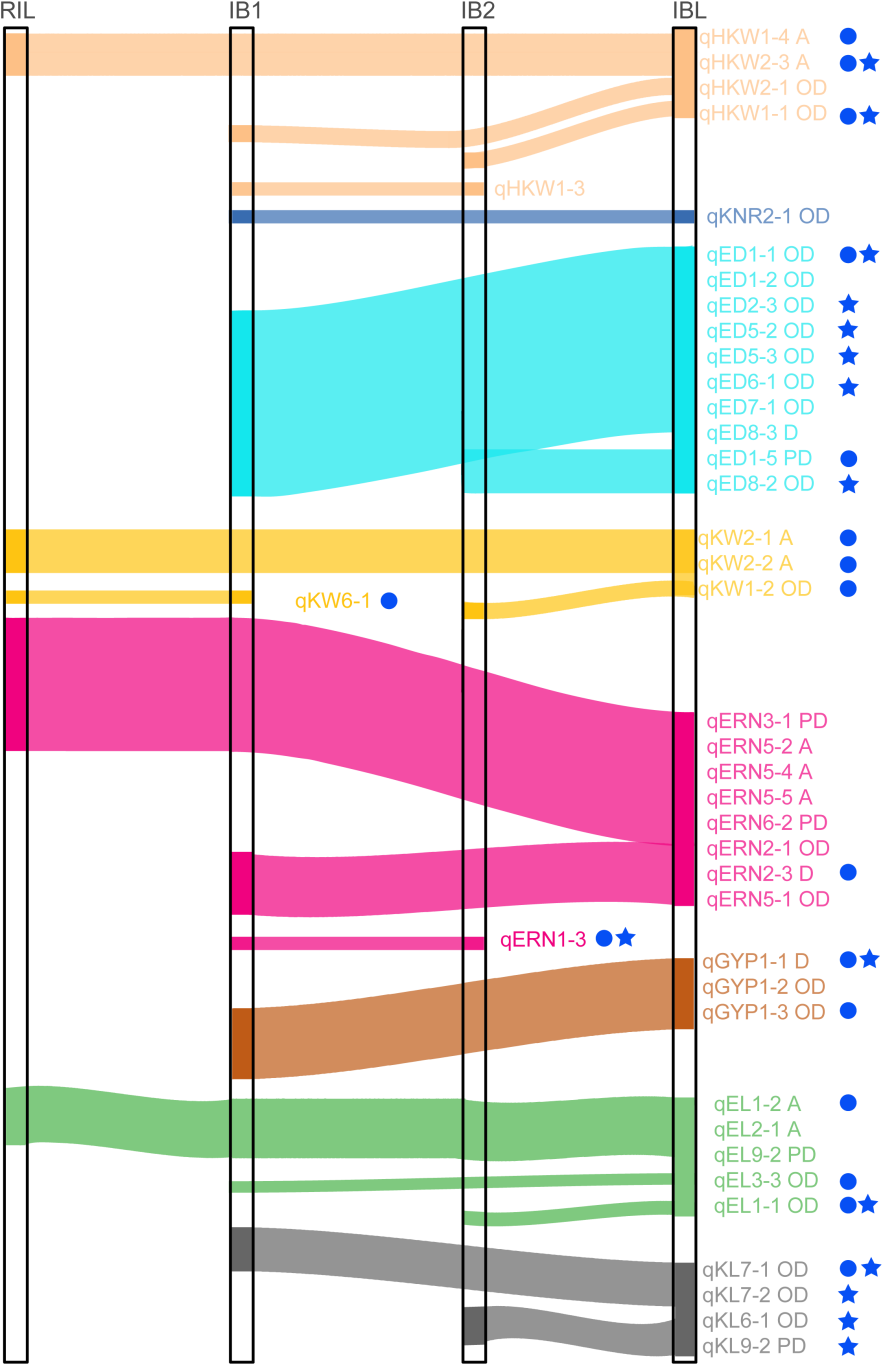
Co-localization of QTLs identified from different populations. Circle represents the QTL with phenotypic variance explained (PVE)>10% in at least one environment. Pentagon refers to stable QTL, which was identified in at least two environments.

Comparison of the QTL results across all populations revealed 41 QTLs were identified in at least two populations. Specifically, 19 QTLs were common to the IB1 and IBL populations, 7 to the IB2 and IBL populations, 12 to the RIL and IBL populations, 1 to the RIL and IB1 populations, three to the IB1 and IB2 populations. (**Figure 4D**). 21 out of these 41 QTLs showed OD effect. Notably, among the QTLs common to the RIL and IBL populations, nine exhibited additive effects and three were partially dominant (**Figure 5**).

### Identification of candidate genes for yield-related traits

**Figure 6:**
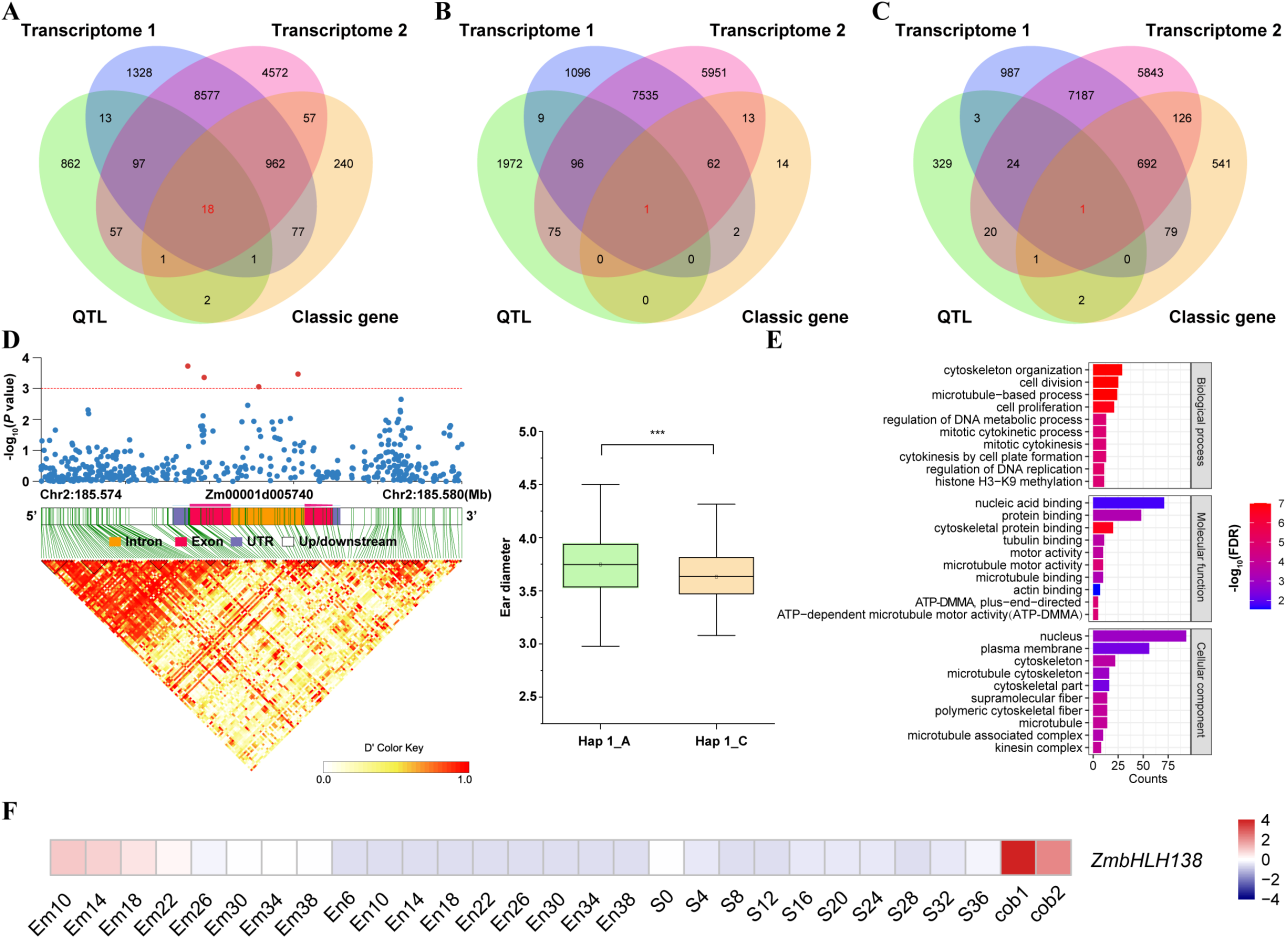
Mining and functional analysis of candidate genes. **(A)** Candidate genes identified for QTLs associated with ear traits. **(B)** Candidate genes identified for QTLs associated with kernel traits. **(C)** Candidate genes identified for QTLs associated with both ear and kernel traits. **(D)** Gene-based association analysis of the candidate gene *Zm00001d005740*. **(E)** Gene Ontology (GO) terms enriched in genes that interact with *Zm00001d005740.* **(F)** The expression abundant of *Zm00001d005740* in different tissues.

Twenty QTLs with PVE > 10% and consistently detected across different environments, populations, or traits were considered key QTLs (**Supplementary Table S3**). All genes located in these key QTL intervals were extracted and compared with two transcriptome datasets, classical yield genes, and their interacting genes. Then, 20 common genes including 18 unique related to ear traits, one unique related to kernel traits, and one shared in ear and kernel traits were saved as candidate genes (**Figures 6A–C**; **Supplementary Table S4**).

To further validate these candidate genes, we performed a gene-based association analysis using 508 high-density genotypic variations and yield trait phenotypes from the traditional association mapping population AM508, which was downloaded from www.maizego.org. For the candidate gene *Zm00001d005740* annotated as bHLH-transcription factor 138 (*ZmbHLH138*), 4 out of the 849 polymorphic loci were significantly associated with ED, with a peak *P*-value (*P* = 1.88E - 04) located in the 5’UTR (**Figure 6D**). Notably, *ZmbHLH138* was most highly expressed in cob (**Figure 6F**), and this supported the association results. Furthermore, the interacting network for *ZmbHLH138* were investigated and 203 interacted genes were identified (**Supplementary Figure S2**; **Supplementary Table S5**). By GO enrichment analysis, we identified 205 significant GO terms, with 185 related to ‘biological process’, 10 to ‘molecular function’, and 10 to ‘cellular component’. The main GO terms included cell division, microtubule-based process, regulation of DNA replication, cytoskeletal protein binding, microtubule motor activity, ATP-dependent microtubule motor activity, tubulin binding, and protein binding, etc (**Figure 6E**; **Supplementary Table S6**). Based on the above analysis, *ZmbHLH138* emerges as a promising candidate gene implicated in the regulation of maize yield traits, particularly in ED. These results warrant further investigation into the functional role of *ZmbHLH138* and its potential applications in breeding programs.

## Discussion

### Overdominance is common in grain yield component trait QTL

For QTL mapping, the traditional genetic population types are F_2_, RIL, backcross population, immortalized F_2_, nested association mapping and doubled haploid population, each of them has some advantage and disadvantage due to detected genetic effects, development time and genetic stability. Also, there were some novel populations developed to address some of the limitations of traditional mapping populations, such as multi-parent advanced generation inter-crossing (MAGIC), complete-diallel plus unbalanced breeding-derived inter-cross (CUBIC) and tested population derived by crossing the RILs with no-parental inbred line (Pascual et al., 2015; Xiao et al., 2021; He et al., 2023). All of these have the common advantage of higher genetic diversity. Our IBL population is named using RIL population and IB population, which is developed by crossing RILs with their respective parents. Traditional RIL populations primarily detect QTLs with additive effects, whereas fixed backcross populations (IB1 and IB2) are capable of identifying QTLs with a-d and a+d effect. Integrated analyses of RIL and fixed backcross populations allow for a more comprehensive assessment of QTL effects in hybrids, facilitating the understanding of their mode of action. Furthermore, the IB1 and IB2 populations offer the advantage of repeatability across multiple environments, which enhances the accuracy of QTL identification (Huo et al., 2023).

Here, we conducted a comprehensive evaluation of yield-related traits using the RIL, IB1, IB2 and combined population IBL in four environments. We detect 121 unique yield-related QTLs. Among them, 42 QTLs were detected in the IBL population and four QTLs are unique to the IBL population, including *qHKW1-7*, *qED3-2*, *qED3–3* and *qKL4-1*. Additionally, we found OD is the main effect for ED, GYP, HKW and KL. There is a complex genetic basis for EL and ERN due to D, OD, PD and A effects. We detected the most QTLs for ED, with a total of 12 QTLs, while KNR had the lowest detection efficiency, with only one QTL. In general, The IBL is a powerful population for dissecting the genetic structure of yield-related traits.

Historically, exploiting heterosis has been crucial for achieving high crop yields (Guo et al., 2014; Labroo et al., 2021). Maize, which is widely utilized in agriculture through single crosses, exhibits yield traits that are predominantly determined by heterosis. By exploring QTLs associated with these traits and deciphering their mechanisms of action, we can significantly enhance maize yield through genetic improvement. In our analysis of the eight yield traits within the IBL population, we found that most QTLs exhibit OD effects. This finding highlights the significant role of OD effects in F_1_ yield and underscores the robust heterosis between parental lines. Exploring additional yield-related QTLs and employing molecular markers to stabilize heterosis loci represent effective strategies for enhancing yield traits and developing high-yield maize varieties.

### Pleiotropic QTLs were common in the component of grain yield

Maize yield is influenced by the complex interplay of multiple traits, such as EL, ED, ERN, KNR, KL, KW, GYP, and HKW. As components of grain yield, there were high positive correlation between most traits. The heritability (*H^2^*) of each trait ranged from 0.51 to 0.89 across the RIL, IB1, and IB2 populations, highlighting variability among traits and populations (**Supplementary Table S1**). Despite these differences, QTL mapping across multiple environments identified only 18 stable QTLs (**Supplementary Table S3**), implying that population background and environmental factors substantially influence yield traits. However, the consistent high heritability observed across populations underscores a predominant genetic control over yield. Therefore, investigation into yield-related loci and their genetic mechanisms remains crucial for enhancing maize yield.

In this study, 121 unique QTLs were distributed across all chromosomes, with chromosome 1 harboring the highest number of QTL and chromosome 10 the fewest (**Figure 3**). However, the distribution of QTLs varies by trait. For instance, QTLs influencing GYP and HKW are predominantly located on chromosome 1, whereas QTLs affecting ERN are mainly found on chromosome 5. Additionally, some QTLs are closely clustered; For example, *qED2–1* and *qED2–2* are within a 0.63 Mb interval on chromosome 2. Similarly, the QTLs such as *qERN3-3*, *qERN3-4*, and q*ERN3–5* tightly clustered between 213.47-229.39 Mb on chromosome 3, whereas *qHKW1-4*, *qHKW1-5*, and *qHKW1–6* are concentrated between 262.78-269.09 Mb on chromosome 1. These QTL clusters suggest that there may be QTL hotspot regions for yield traits on the chromosomes. However, further fine mapping and functional validation are needed to distinguish possibility. Notably, 26 QTLs with PVE greater than 10% were detected in the RIL, IB1, and IB2 populations, respectively. But, the highest PVE observed among QTLs in the IBL population was only 6.11%, which could be due to the combined genetic background affecting trait variance, although other factors such as environmental interactions or statistical power may also play a role.

By compared those QTLs across different populations, we identified 41 QTLs shared in different groups (**Figure 4D**). Specifically, 38 QTLs from those QTLs identified in the IBL population were shared that in the RIL, IB1, and IB2 populations. These co-localized QTLs across populations are potential candidates for future functional gene cloning and molecular marker development due to their stability. Furthermore, *qGYP1–1* and *qED1–1* are both located at 8.94-15.81 Mb on chromosome 1, while *qGYP1–7* and *qERN1–3* are both located at 294.82-296.09 Mb on chromosome 1. Additionally, *qGYP1-4*, *qED1-3*, and *qEL1–1* are all located on chromosome 1 at 224.14-224.98 Mb (**Supplementary Table S3**). The co-localization of these QTLs suggests the possibility of pleiotropic or tightly linked genes influencing multiple traits.

When comparing QTL results with those from previous studies, we observed several notable overlaps. For instance, *qERN5–2* and *qERN5–3* were located within the *qERN5–*2 interval previously identified from the F_5:6_ RIL population derived from KA105 and KB020, showing positive additive effects from KA105 (Yang et al., 2021). Similarly, *qED1-1*, characterized by an overdominance effect in our study, coincides with the previously detected *qED1–*1 in a RIL population (Chen et al., 2016). In addition, we found that the candidate gene *Zm00001eb217930* for ERN is located within *qERN5-1* (Shen et al., 2019).The *Zm00001eb061800* that affects kernel weight is located within *qGYP1-5*, and these genes may be functional genes within these QTLs (Wang et al., 2022).

### *ZmbHLH138* participated in regulating the component of grain yield

The basic helix-loop-helix (bHLH) transcription factors are widely distributed among eukaryotes and constitute the second-largest family of transcription factors in plants, following the MYB family (Feller et al., 2011; Gyoja, 2017). These factors play roles in various processes, such as plant growth, secondary metabolite metabolism, and responses to abiotic stress (Penfield et al., 2005; Seo et al., 2011; Ohno et al., 2013; Liu et al., 2014; Qi et al., 2014). In maize, *ZmbHLH55* improves salt tolerance by increasing the accumulation of ascorbic acid (Yu et al., 2021), while Opaque11 regulates endosperm development and affects starch and protein content (Feng et al., 2018). In this study, *Zm00001d005740* was identified within the *qERN2–3* intervals from both the IB1 and IBL populations. Additionally, gene-based association analysis revealed 4 polymorphic loci that are significantly associated with ED (**Figure 6D**). Moreover, *Zm00001d005740* shows high expression in ears, and its interacting gene has been identified as a candidate gene related to yield. These findings suggest that *Zm00001d005740* potentially improves yield by influencing ED.

## Conclusion

In this study, QTL mapping was conducted for yield-related traits across various populations, and 121 unique QTLs were identified. The majority of QTLs (59.5%) in the IBL population exhibited overdominance, which suggested the yield-related traits in this population were mainly regulated by overdominance. By combining the public transcriptome data, twenty genes were considered as candidate genes for yield-related traits, among which *Zm00001d005740* was significantly correlated with ear diameter. The annotation of the interacted genes with *Zm00001d005740* showed that this gene may regulate yield through the cell division biological pathway. These research not only advances our understanding of maize yield but also provides a gene source for future work in functional gene cloning and the development of high-yield maize varieties through selective breeding.

## Data Availability

The datasets presented in this study can be found in online repositories. The names of the repository/repositories and accession number(s) can be found in the article/**Supplementary Material**.
