# Supplementary figures and images for: Dissecting the genetic architecture of yield-related traits by QTL mapping in maize
Source: Front Plant Sci. 2025 Aug 15;16:1624954. doi: 10.3389/fpls.2025.1624954 (PMC12394503; doi:10.3389/fpls.2025.1624954)

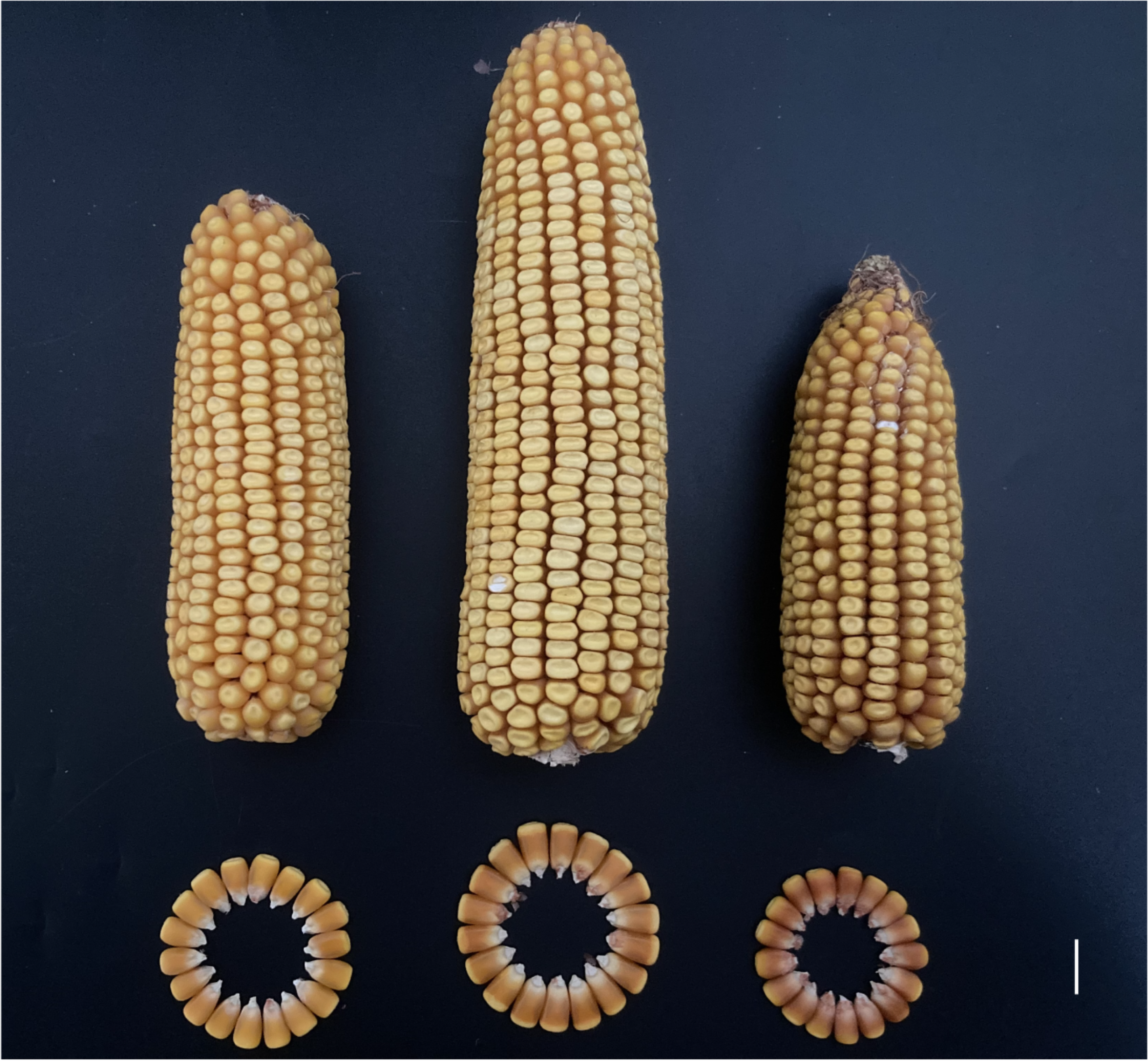

Supplement: Supplementary file 1 [file Image1.tif]

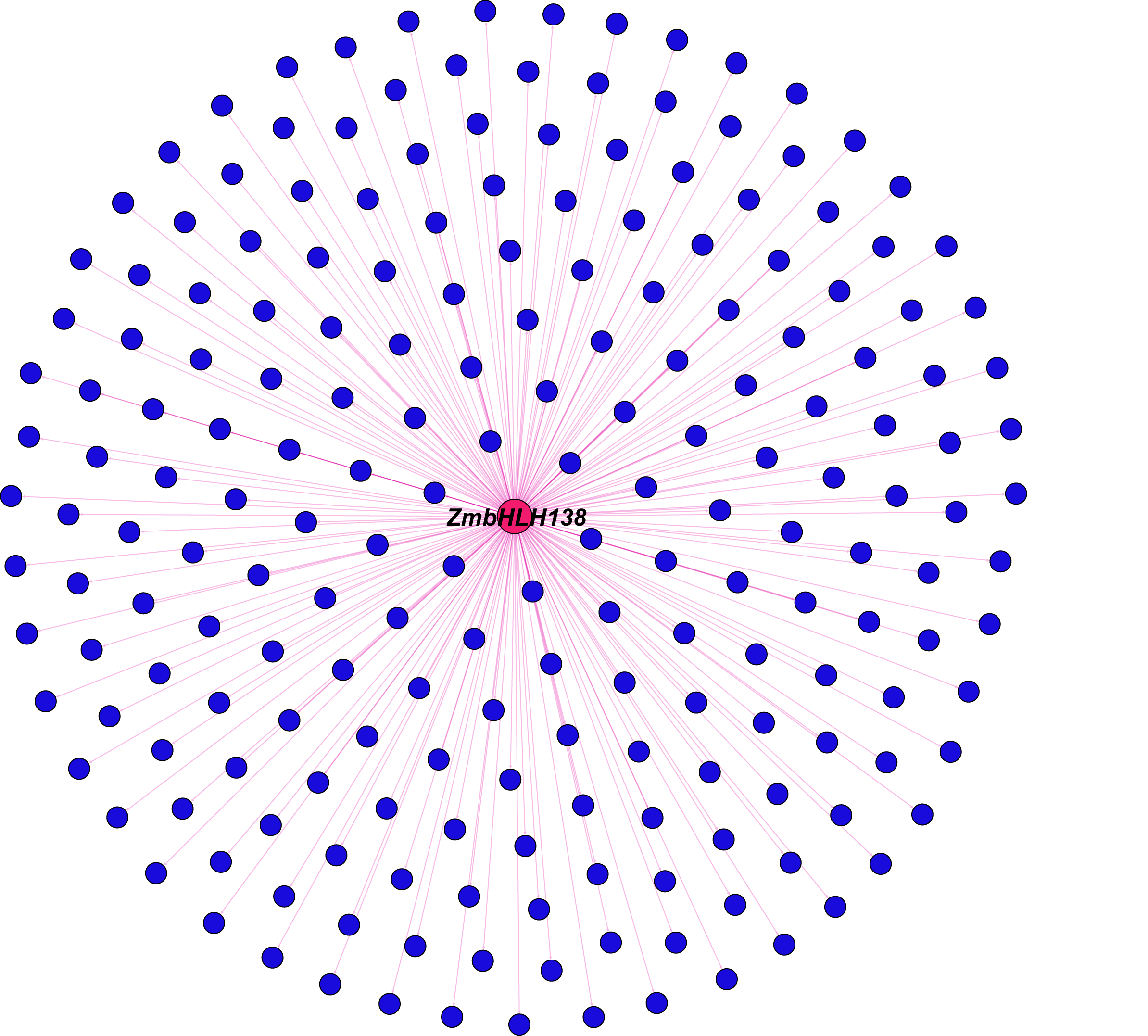

Supplement: Supplementary file 2 [file Image2.tif]
